# Supplementary material for: Salivary gland ultrasound is associated with the presence of autoantibodies in patients with Sjögren’s syndrome: A Danish single-centre study
Source: PLoS One. 2022 Dec 12;17(12):e0265057. doi: 10.1371/journal.pone.0265057 (PMC9744271; doi:10.1371/journal.pone.0265057)
Supplement: S1 File — (PDF) [file pone.0265057.s001.pdf]

Age	Disease duration (months)	Sex	ANA pos	Anti-Ro52	Anti-Ro60	Anti-La	Reumafactor titer	ESSDAI	ESSPRI
64	86	2	1				300	12	21
73	443	2	1	1685.0	1375.0	63.0	17	22	14
77	70	2	1	1685.0	1375.0	692.0	300	0	25
70	389	2	1	1502.0	1375.0	461.0	50	11	24
65	74	2	1	0.0	1062.0	1550.0	25	11	18
55	9	2	1	1685.0	1375.0	78.0	45	2	19
60	9	2	1	0.0	219.0	0.0	26	2	12
59	145	2	1	539.0	1375.0	411.0	0	3	16
74	16	2	1	209.0	1375.0	0.0	39	7	22
71	168	2	0				0	17	28
55	51	2	1				24	2	23
55	5	2	1	0.0	1375.0	0.0	24	1	24
63	33	2	1	141.0	0.0	0.0	0	2	23
41	16	2	1	1172.0	0.0	0.0	50	2	21
48	23	2	1	40.0	1375.0	1550.0	0	7	20
35	60	2	1	510.0	1115.0	880.0	300	7	24
63	36	2	1	1325.0	1375.0	23.0	300	3	17
57	36	2	1	1121.0	1375.0	0.0	55	3	28
41	66	2	1	1685.0	1375.0	1550.0	300	26	19
67	31	1	1	198.0	0.0	0.0	0	13	13
54	28	2	0				0	15	24
53	17	2	0				0	2	12
56	247	1	1	701.0	1375.0	127.0	66	0	10
50	198	2	1	1685.0	1375.0	1550.0	300	12	15
59	118	2	1	0.0	616.0	0.0	0	2	26
61	143	2	1				0	5	26
52	18	2	0				0	11	25
50	93	2	1	1685.0	1375.0	0.0	297	11	24
64	162	2	1				50	2	20
53	46	2	1	1685.0	1375.0	1550.0	40	18	23
59	103	2	0				0	5	17
58	16	2	1				0	2	30
62	30	2	1	98.0	0.0	0.0	0	7	15
66	90	2	1	171.0	0.0	0.0	33	4	23
65	390	2	1	1685.0	1375.0	325.0	209	3	11
59	102	2	0				0	6	24
53	186	2	1				0	2	22
53	48	2	1	1685.0	1375.0	109.0	203	13	24
76	89	2	1				44	6	21
71	180	2	1	1685.0	1375.0	1384.0	38	16	25
70	8	2	1	1685.0	1375.0	186.0	36	18	22
66	5	2	1	1685.0	1375.0	903.0	300	16	26
48	21	2	0	0.0	0.0	0.0	0	5	17
73	38	2	1	25.0	20.0	0.0	145	0	14
57	176	2	1	1685.0	1375.0	720.0	300	0	9
53	6	2	1	1685.0	1375.0	39.0	245	3	18

75	144	2	1	200.0	600.0	1550.0	43	2	23
85	84	2	1	1685.0	1375.0	1550.0	300	4	12
68	104	2	1	1685.0	1375.0	96.0	83	9	21
