# Supplementary material for: Salivary gland ultrasound is associated with the presence of autoantibodies in patients with Sjögren’s syndrome: A Danish single-centre study
Source: PLoS One. 2022 Dec 12;17(12):e0265057. doi: 10.1371/journal.pone.0265057 (PMC9744271; doi:10.1371/journal.pone.0265057)
Supplement: Attachment — Submitted filename: Letter to editor13.12.21_AV_30HLi.docx [file pone.0265057.s002.docx]

PONE-D-21-25023

Letter to editor

Journal requirements:

1.	Checked

2.	p 6, Line 142 ”unpublished data” has been adjusted to reference 19 – an abstract, where	data is published. The manuscript is in preparation

2.	We have clarified the justification for the recruitment and sample size in the study. Sample size was calculated under the assumption of background prevalence of 51% (49% of the patients recently diagnosed pSS had SGUS at 0 or 1 as indicated by reference 17), expected 40% higher frequency of SGUS abnormality among our patients previously diagnosed with pSS, Alpha 005, Beta 0.2, Power 0.8. Suggested sample size 44. Consecutive patient were included

p 5, line 114-118 Patients followed at Odense University Hospital with the diagnosis pSS	based on the AECG criteria of 2002 and with appointments in the patient clinic in the	period November 2019 to February 2020 (108 patients) were invited to participate in the project. In all, 49 consecutive patients were included (45.4 %) in a cross-sectional study.

And line 120 No information about non-participants is available.

3.	Additional details regarding participant consent.

p 6, line 123-124 All Patients gave informed consent after verbal and written information	according to the approval.

4.+5.	Funding information section and financial disclosure has been aligned. And grant number has	been added

Desired Update to Funding information section (Has been copied to the cover letter, so it	can be changed the online submission. Thank you for helping us with this issue)

The study was supported by the Danish Rheumatism Association. Grants R181-A6347 and	R178-A6346

Reviewer´ comments:

Question 1 to 4: Thank you. We have no further comments

Q 5:

#1	1. Thank you for the suggestions. The title has been adjusted according to the suggestion.	page1 line 5-6: Salivary gland ultrasound is associated with the presence of autoantibodies	in patients with Sjögren´s Syndrome: A Danish single-centre study

2. Thank you for addressing this point. We have explained focus score in the manuscript. Page 4 Line 89-90. The Salivary gland (SG) function and pathology can be characterized, as suggested in the 2002 AECG criteria, through minor SG biopsy quantifying a focus score (defined as infiltrates of mononuclear cells with at least 50 cells pr 4 mm^2^) and assessing unstimulated	salivary flow.

3.	Thank you. This is logical. The number the has been swapped in text and table

#2

1. SGUS abnormalities are irreversible changes of the salivary glands, hence, disease duration and age are associated with more severe findings as illustrated in table 1, page 11. Disease duration has been added to the table and higher SGUS scores are associated with longer disease duration.

Our cross-sectional study design does unfortunately not allow us to determine the time in the disease where SGUS abnormalities are developed.

Comments have been added p 15, line 303-7 inclusive 2 new references to support the statements. The pathogenesis of pSS is multifactorial with important contributions from genetic susceptibility, infection and local inflammation (29). The B-cells are central in this process linking local inflammation (“overactive” B-cells) in the salivary glands with the systemic synthesis of autoantibodies (30).

2.We do agree that the outcome measure is not sensitive/optimal, but also as stated by the	reviewer these are currently the only available. Comments has been added p. 15 line 322-	323 for a possible explanation: Correspondingly, SGUS findings did not reflect the patient	reported ESSPRI where extra glandular symptoms account for the most domains.

3.Please see our comment above

4.We do appreciate this very interesting comments/question. Presently we do not have any data or knowledge to qualify this point. Further projects should look into this.

5.	Thank you for the comment. The authors find it very import in this relative new research area to address the literature and the comparability of our Danish cohort other cohorts.
